# Three cases of nonmetastatic prostate cancer treated successfully with primary intermittent androgen deprivation therapy over 10 years

**DOI:** 10.1002/ccr3.854

**Published:** 2017-02-23

**Authors:** Yohei Shida, Tomoaki Hakariya, Yasuyoshi Miyata, Hideki Sakai

**Affiliations:** ^1^ Department of UrologyNagasaki University Graduate School of Biomedical Sciences1‐7‐1 SakamotoNagasaki852‐8501Japan

**Keywords:** Androgen deprivation, continuous androgen deprivation, intermittent androgen deprivation, nonmetastatic, prostate cancer

## Abstract

We report three cases of nonmetastatic prostate cancer treated effectively with long‐term primary intermittent androgen deprivation (IAD). IAD is not a standard therapy for patients with nonmetastatic prostate cancer. However, based on our experience, we suggest that IAD is one of useful therapeutic tools under certain patients’ condition.

## Introduction

Primary hormone therapy has not been recommended for men with localized or locally advanced prostate cancer as a standard therapy in Western countries. The European Association of Urology (EAU) guidelines state that androgen suppression is not suitable for low‐risk cancer. It also states that androgen suppression monotherapy is “no place in asymptomatic patients” for intermediate‐risk cancer. For high‐risk cancer, it recommends reserving for patients unwilling or unable to receive any form of local treatment. They are either symptomatic or asymptomatic with a prostate specific antigen (PSA) doubling time <12 months and a PSA > 50 ng/mL and a poorly differentiated tumor 1.

The Japanese Urological Association reported that 45% of clinical T1c‐T3 cases had had primary hormone therapy in Japan 2. Akaza et al. 3. reported that the progression of prostate cancer was retarded by primary hormone therapy in men with localized or locally advanced prostate cancer. They concluded that the men who had been treated for prostate cancer with primary hormone therapy or prostatectomy had a life expectancy similar to that of the normal population. Thus, it has been suggested that Japanese men with localized or locally advanced prostate cancer have a possibility of receiving an advantage of hormone therapy.

However, the efficacy and safety of IAD for the Japanese men with localized or locally advanced prostate cancer is still largely unknown. In this report, three cases of localized or locally advanced prostate cancer treated with IAD are described. All of the treatment was initially induced with combined androgen blockade (CAB) for a primary hormone therapy and was ceased at a PSA < 0.1 ng/mL. Trigger for restart hormone therapy was a PSA level between 1 and 2 ng/mL. Radiographic monitoring including computed tomography (CT) and bone scintigraphy were performed before restarting hormone therapy.

## Case Histories

### Patient 1

**Figure 1:**
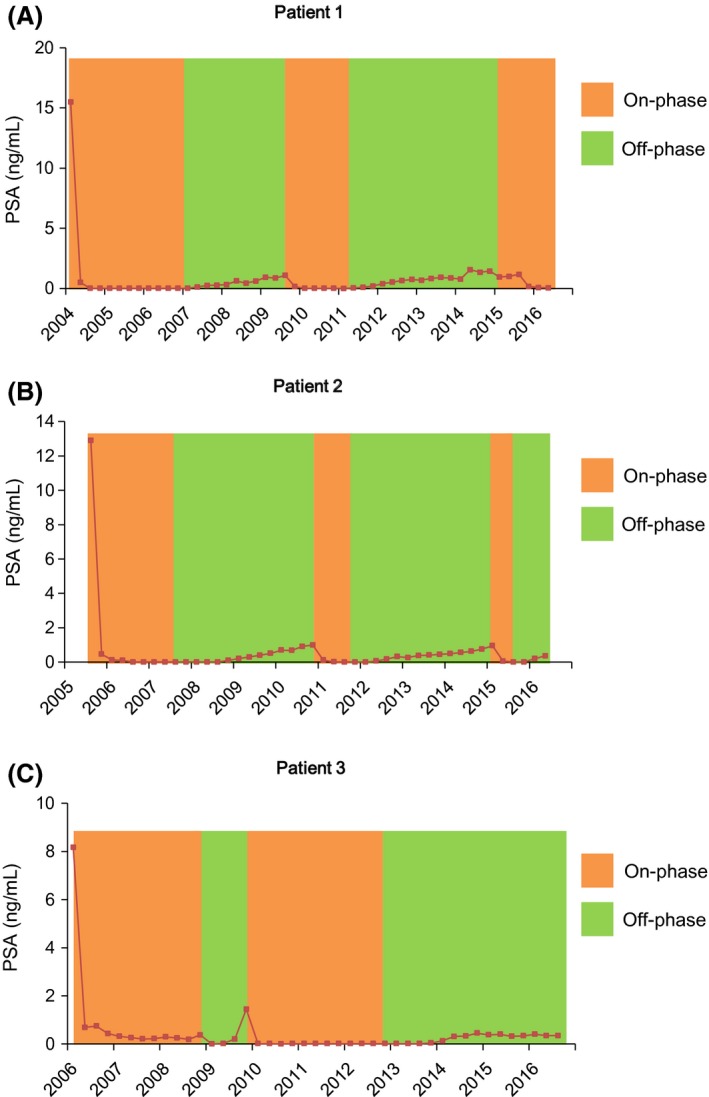
PSA course of three cases. Colors illustrate therapy phases (orange: on‐phases; green: off‐phases). (A) Patient 1: on‐phases 65/144 months (45.1%); off‐phases 79/144 months (54.9%). (B) Patient 2: on‐phases 48/129 months (37.2%); off‐phases 81/129 months (62.8%). (C) Patient 3: on‐phases 63/127 months (49.6%); off‐phases 64/127 months (50.4%). PSA, prostate specific antigen.

A 71‐year‐old man presented with a PSA elevation of 15.5 ng/mL. After diagnosis of locally advanced prostate cancer (cT3bM0M0) of Gleason 7 (3 + 4), he started CAB with leuprorelin and bicalutamide in July 2004. A total of off‐phases were 54.9% (79/144 months) (Fig. 1A). To date, the patient has no clinical and radiological sign of progression. The patient is again in on‐phase since January 2015 and the latest PSA level was 0.05 ng/mL.

### Patient 2

A 77‐year‐old man presented with a PSA elevation of 12.9 ng/mL. After diagnosis of localized prostate cancer (cT2aN0M0) of Gleason 8 (5 + 3), he started CAB with goserelin and chlormadinone acetate in September 2005. A total of CAB on‐phases were 19.4% (25/129 months) (Fig. 1B). Depending on the wishes of the patient, CAB was changed to the monotherapy of bicalutamide 80 mg orally once daily from a second on‐phase. A total of bicalutamide on‐phases were 17.8% (23/129 months). To date, the patient has no clinical and radiological sign of progression. A total of off‐phases were 62.8% (81/129 months). The patient is again in off‐phase from December 2015 and the latest PSA level was 0.37 ng/mL.

### Patient 3

A 75‐year‐old man presented with a PSA elevation of 8.17 ng/mL. After diagnosis of localized prostate cancer (cT1cN0M0) of Gleason 9 (4 + 5), he started CAB with goserelin and chlormadinone acetate in January 2006. A total of on‐phases were 49.6% (63/127 months) (Fig. 1C). CAB was changed to the monotherapy of goserelin from a second on‐phase. The patient is continuing off‐phase from August 2012. To date, the latest PSA level was 0.35 ng/mL and the patient has no clinical and radiological sign of progression.

## Discussion

The discussion on long‐term primary continuous androgen deprivation (CAD) and primary IAD for men with localized or locally advanced prostate cancer is controversial because some of the patients receive much benefit from these therapies. Labrie et al. 4 reported that long‐term primary hormone therapy might be curative for men with localized prostate cancer. They reported that only 10% of patients with localized prostate cancer who received CAB for >6.5 years demonstrated PSA relapse at 5 years after cessation of CAB. On the other hand, 64% of patients who received CAB for 3.5–6.5 years experienced PSA relapse. However, primary hormone therapy is generally considered palliative for patients with localized prostate cancer who are unwilling or unable to receive radical treatment 1. Current guidelines state that IAD is recommended or considered for men with recurrence after radical therapy (Table 1). Although there has been increasing interest in IAD, the current literature is still limited, especially for nonmetastatic prostate cancer. In a phase 3b randomized study, IAD and CAD demonstrated similar efficacy, tolerability, and quality of life (QOL) in patients with relapsing M0 and locally advanced prostate cancer. However, there were no apparent QOL benefits. It also stated that the principal benefit of IAD compared with CAD is a potential cost reduction with comparable overall survival (OS) rates 5.

**Table 1:** Recommendation on intermittent androgen deprivation therapy

Year	Organization	Recommendation
2016	EAU 1	Intermittent ADT might be an option with metastatic disease after a standard induction period.
2015	ESMO 9	Intermittent ADT is recommended for men with biochemical relapse after radical RT starting ADT. Continuous ADT is recommended as first‐line treatment of metastatic, hormone‐naïve disease.
2015	NCCN 10	ADT for biochemical recurrence; men who choose ADT should consider intermittent ADT.

EAU, The European Association of Urology; ESMO, the European Society For Medical Oncology; NCCN, The National Comprehensive Cancer Network; ADT, androgen deprivation therapy; RT, radiation therapy.

In the present cases, one of the reasons for selecting IAD was the treatment cost. They were initially induced with CAB for a primary hormone therapy and was ceased at a PSA < 0.1 ng/mL. Timing to restart hormone therapy was set at a PSA level between 1 and 2 ng/mL. PSA level for ceasing or restarting hormone therapy was set at comparatively lower level than other studies. They were effectively treated with IAD for more than 10 years at a cost of <5 years. Although all patients correspond to high‐risk cancer of D'Amico classification, they received CAB for <5 years and demonstrated no PSA relapse, and no clinical and radiological sign of progression. Additionally, all patients did not experience any serious side effects. Thus, even though classified in high‐risk prostate cancer, primary IAD might be valid for long periods in some patients. Based on these three cases, we suspect that IAD is worth discussing as an initial therapy in patients with nonmetastatic prostate cancer. De Leval et al. 6. suggested that patients with nonmetastatic and poorly differentiated cancer benefit most. IAD should be selected with caution because some studies have reported lower median survival 7. Patients who clearly do not benefit from IAD are patients with symptomatic high‐burden disease and high initial PSA levels 7, 8. To identify the characteristics of the patients who would benefit from IAD, further studies are necessary.

## Consent

Written informed consent was obtained from the patients.

## Authorship

YS: contributed to the conception, design, and drafting of the manuscript and wrote the manuscript. TH: contributed to the critical revision of the manuscript. YM: contributed to the writing and critical revision of the manuscript. HS: contributed to the design and critical revision of the manuscript. All authors read and approved the final manuscript.

## Conflict of Interest

The authors declare that they have no competing interests.
